# Access to Medicines in Bulgaria and North Macedonia: Legislative, Pricing, and Reimbursement Perspectives

**DOI:** 10.3390/pharmacy14020052

**Published:** 2026-03-23

**Authors:** Anna Todorova, Dijana Miceva, Mariya Ivanova, Tanya Kazakova, Bistra Angelovska

**Affiliations:** 1 Faculty of Pharmacy, Medical University Varna, 9000 Varna, Bulgaria; anna.todorova@mu-varna.bg (A.T.); tanya.kazakova@mu-varna.bg (T.K.); 2 Faculty of Medical Sciences, Goce Delcev University, Krste Misirkov Str., No. 10-A, P.O. Box 201, 2000 Stip, North Macedonia; dijana.miceva@ugd.edu.mk (D.M.); bistra.angelovska@ugd.edu.mk (B.A.)

**Keywords:** community pharmacies, pricing, prescribing, pharmacists, pharmaceutical legislation, reimbursement, EU, Bulgaria, North Macedonia

## Abstract

National legislative frameworks governing prescribing, pricing, reimbursement, and dispensing play a decisive role in shaping access to medicines. This study examines the financial availability of medicines in Bulgaria and North Macedonia through a comparative review of national pharmaceutical legislation, pricing mechanisms, reimbursement models, and digitalisation policies, assessed in relation to European Union standards. The findings indicate that access to medicines in both countries is shaped by the combined effects of multiple regulatory and financial instruments rather than by individual policy measures. Both systems apply strict control of prescribing and dispensing, external reference pricing, and positive reimbursement lists, reflecting alignment with international recommendations. However, significant differences in policy design lead to divergent access outcomes. Bulgaria’s more advanced digitalisation of prescribing and reimbursement, including mandatory electronic prescribing for selected therapeutic groups, enhances regulatory oversight and expenditure control but is associated with higher patient out-of-pocket expenditure, partly due to the application of the standard value-added tax on medicines. In contrast, North Macedonia combines lower taxation with capped patient co-payments, higher regulated pharmacy margins, and fixed pharmacy remuneration per prescription, contributing to improved financial affordability for patients while supporting pharmacy sustainability. Additional instruments, such as the Generics without Co-Payment List, further strengthen patient financial protection. The study provides comparative evidence relevant to pharmaceutical policy reforms and highlights the importance of balanced regulatory approaches that promote affordability, system sustainability, and equitable access to medicines.

## 1. Introduction

Access to medicines is widely recognised as a fundamental component of effective health systems and a key determinant of population health outcomes [1,2]. The availability, affordability, and appropriate use of medicinal products are strongly influenced by national legislative frameworks governing the prescribing and dispensing of medications [1]. Patients’ access to medicines is directly influenced by national pharmaceutical policies and regulatory regimes, including pricing and reimbursement mechanisms that shape the availability of medicines within primary healthcare systems [2].

The World Health Organization (WHO) identifies transparent pricing policies, sustainable reimbursement mechanisms, and strong regulatory governance as essential prerequisites for equitable access to essential medicines [3]. WHO guidelines emphasise the importance of external reference pricing, generic medicine policies, and demand-side measures to improve affordability while maintaining health system sustainability [4]. Within the European Union (EU), pharmaceutical policy initiatives increasingly focus on balancing patient access, financial sustainability, and incentives for innovation. The EU Pharmaceutical Strategy highlights affordability, security of supply, and digital transformation as key priorities for strengthening pharmaceutical systems across Member States and neighbouring countries [5,6].

Within Southeast Europe, Bulgaria and North Macedonia represent two closely connected healthcare systems operating under different institutional and regulatory conditions. In Bulgaria, pharmaceutical policy constitutes an integral part of the National Health Strategy and is aligned with the health and pharmaceutical policies of the European Union (EU) [7,8]. Within this framework, the registration of medicines follows EU regulatory procedures, enabling earlier market entry of newly authorized medicinal products [9]. Within this framework, the registration of medicines follows EU regulatory procedures, enabling earlier market entry of newly authorized medicinal products. The national system incorporates mechanisms for price regulation, external reference pricing, and reimbursement of medicines, as well as the implementation of health technology assessment (HTA) for new therapies [10]. These measures aim to reduce financial risk and improve cost-effectiveness within the public healthcare system [3,11].

North Macedonia, in contrast, is an EU candidate country undergoing gradual alignment with European pharmaceutical policy principles. However, medicine registration remains predominantly based on national authorization procedures, which may prolong administrative timelines and delay the availability of certain medicines compared to EU Member States. These regulatory characteristics can result in later patient access to innovative or specialized therapies. While maintaining national regulatory autonomy, the country has introduced pricing and reimbursement policies influenced by WHO and EU recommendations, including reference pricing and regulated margins for medicines reimbursed by the national health insurance fund [12,13]. Differences in institutional structures, financing arrangements, and regulatory maturity between the two countries create distinct approaches to medicine access and affordability, despite their geographical proximity and shared historical characteristics of healthcare system development.

The transition towards digital health systems in both countries coincided with the COVID-19 pandemic, which exposed structural weaknesses in pharmaceutical supply chains and accelerated the adoption of electronic prescribing to ensure continuity of care and uninterrupted access to therapies for patients with chronic conditions [10,14].

National health policies also shape the financial affordability of medicines. Key influencing factors include pricing regulations, reimbursement mechanisms, generic medicine policies, and broader socioeconomic determinants [4,10].

Bulgaria faces significant demographic and epidemiological challenges, including population ageing and an increasing prevalence of chronic diseases. These trends have led to growing demands for investment in the healthcare sector and the need for strict regulation of public pharmaceutical expenditure [10]. The National Council on Prices and Reimbursement of Medicinal Products serves as the central institutional body responsible for regulating this area, promoting transparency and price control, including the development of publicly accessible applications providing information on maximum regulated medicine prices in national currency and euros [15].

North Macedonia, in contrast, is experiencing rising consumption of medicines for chronic conditions alongside a need to improve access to pharmaceutical services, particularly in rural and underserved areas. National registries and decisions on maximum medicine prices, as published by the Ministry of Health and the Central Register of Medicinal Products and Medical Devices, reflect active policy interventions aimed at regulating public expenditure and improving access to essential therapies [16,17].

Despite numerous studies on pharmaceutical pricing and reimbursement policies in individual countries, there is limited comparative evidence on how legislative frameworks governing prescribing and dispensing influence financial access to medicines in Southeast European countries. In particular, comparative analyses between EU Member States and EU candidate countries remain scarce. By contrasting an EU member state (Bulgaria) with a non-EU country (North Macedonia), this study would provide valuable insights into regulatory, digital, and financial factors that facilitate or constrain the availability, affordability, and sustainability of medicines. The findings would be significant for public health policy, particularly in relation to better access to medicines and future harmonization with EU.

### Purpose

The aim of this study is to examine the key characteristics of current legislation governing the financial availability of medicines, and to identify similarities, differences, and regulatory gaps between Bulgaria and North Macedonia in the context of European Union standards.

## 2. Materials and Methods

### 2.1. Study Design

This study employs a comparative case study design to investigate how legislative, pricing, and reimbursement frameworks impact access to medicines in Bulgaria and North Macedonia. A comparative study design is a research approach that systematically examines two or more cases, systems, or policy contexts to identify similarities, differences, and explanatory factors influencing observed outcomes. Such an approach is particularly suitable for analysing national pharmaceutical policy frameworks, as it enables structured comparison of regulatory environments and facilitates interpretation of how different policy instruments influence access to medicines [18,19].

### 2.2. Case Selection

Each country is treated as a distinct policy case. The two countries share geographical proximity and historical similarities in the organisation of their healthcare systems, but different EU status. This allows a meaningful comparison of how varying levels of regulatory harmonisation with European Union pharmaceutical policies influence national approaches to medicine prescribing, pricing, reimbursement, and access. Additionally, both countries have recently implemented legislative changes related to digital prescribing and pharmaceutical financing, making them relevant and timely cases for comparative analysis.

### 2.3. Data Sources

Data were collected through a structured review of publicly available documents and official sources, grouped into four main categories:

1. National legislation—primary pharmaceutical and healthcare laws regulating medicinal products, prescribing and dispensing practices, pricing, reimbursement;
2. Subordinate legislation and regulatory acts, ordinances, rulebooks, methodological guidelines, and ministerial regulations governing the practical implementation of pharmaceutical policy, including prescribing requirements, reimbursement procedures, pricing methodologies, and mark-up regulations;
3. Official registers and institutional sources- data and publications from national regulatory and administrative bodies, including medicines agencies, health insurance funds, ministries of health, and publicly accessible registers related to medicinal products, pharmacies, and pricing;
4. International and supranational documents- policy documents, reports, and strategic frameworks issued by WHO and the European Union.

The identification and collection of documentary sources were conducted collaboratively by all authors. Each researcher contributed to retrieving relevant legislative documents, regulatory acts, and institutional publications from official national and international sources related to pharmaceutical regulation, pricing, reimbursement, and digital health systems. To ensure accuracy and methodological consistency, the collected materials were independently reviewed and cross-checked within the research team. Any differences in interpretation or classification were discussed among the authors and resolved through consensus before inclusion in the comparative analysis.

### 2.4. Inclusion and Exclusion Criteria

Documents were included in the analysis if they:

- Were officially published or in force in either Bulgaria or North Macedonia;
- Directly addressed pharmaceutical regulation, prescribing and dispensing rules, pricing mechanisms, reimbursement policies, or digital health systems;
- Were published or applicable within the period 2007–2024, capturing recent regulatory developments and reforms, including those related to the COVID-19 pandemic.

Documents were excluded if they:

- Focused exclusively on hospital-level procurement or inpatient pharmaceutical financing;
- Addressed clinical effectiveness or therapeutic outcomes without policy or regulatory relevance;
- Lacked official or institutional validation (e.g., non-authoritative sources).

A comparative analytical framework was applied to ensure systematic and consistent examination across both case studies. The analysis focused on the following key determinants:

1. Prescribing rules—legal requirements and procedural rules governing who may prescribe medicines, prescription validity, prescription types, and the use of electronic prescribing systems.
2. Pricing mechanisms—national approaches to medicine price regulation, including external reference pricing, price-setting methodologies, and regulated wholesale and retail mark-ups.
3. Reimbursement policies—criteria and mechanisms for public funding of medicines, including positive lists, reimbursement levels, patient co-payments, and financial arrangements between pharmacies and health insurance funds.
4. Digitalisation—the role of electronic prescribing, digital reporting systems, and health information platforms in prescribing, dispensing, and reimbursement processes.
5. Financial accessibility—policy measures influencing patients’ out-of-pocket payments, including co-payment levels, value-added tax (VAT) on medicines, and other cost-containment or affordability-related mechanisms.

Data were analysed descriptively and comparatively, with similarities and differences between the two countries identified and interpreted in relation to their regulatory environments and institutional contexts. The findings were used to assess how policy design and legislative frameworks contribute to or constrain financial access to medicines in each case.

## 3. Results

The results are presented through a structured comparison of Bulgaria and North Macedonia, focusing on five key policy domains influencing access to medicines: prescribing rules, pricing mechanisms, reimbursement policies, digitalisation, and financial accessibility. The comparative findings are summarised in tables, while the narrative highlights the most relevant similarities and differences between the two cases.

### 3.1. Prescribing Rules and Dispensing Requirements

Table 1 presents a comparison of prescribing and dispensing regulations in Bulgaria and North Macedonia, including prescription types, validity periods, prescribing authority, and rules for chronic and acute therapies.

The analysis shows that both countries maintain strict legislative frameworks governing prescribing, but differ in their operational design. In Bulgaria, prescribing is increasingly digitalised, with mandatory electronic prescriptions for specific therapeutic groups such as antidiabetic medicines and systemic antibiotics. Prescription validity varies depending on the prescription type, with distinct rules for single and repeat dispensing.

In North Macedonia, prescriptions are primarily issued through an e-prescribing system operated by the national health insurance fund, but paper prescriptions are still required for acute treatments. Prescriptions for acute conditions have a shorter validity period, while medicines for chronic conditions may be prescribed for extended treatment periods, subject to monthly dispensing limitations. In both countries, medicines containing narcotic substances are subject to enhanced control and reporting requirements, reflecting harmonisation with international regulatory standards. In North Macedonia, prescriptions for narcotic medicinal products included in the Positive List must be explicitly marked with the designation “N” (narcotic). Such prescriptions are valid for five days from the date of issuance, after which dispensing is not permitted.

### 3.2. Pricing Mechanisms and Mark-Up Regulation

Pricing mechanisms in Bulgaria and North Macedonia differ in terms of reference country baskets, mark-up calculation bases, and VAT application (Table 2). Table 2 compares national pricing mechanisms, reference pricing systems, and regulated wholesale and retail mark-ups.

Both countries apply external pricing reference as a core principle for setting medicine prices. However, differences are observed in the selection of reference countries, price calculation methodologies, and mark-up structures. Bulgaria applies regulated wholesale and retail mark-ups based on the manufacturer price, with upper monetary limits for higher-priced medicines. In contrast, North Macedonia applies mark-ups primarily based on wholesale prices, with higher percentage margins for lower-priced medicines.

These structural differences contribute to variations in price formation and affect both pharmacy remuneration and final patient prices.

### 3.3. Reimbursement Policies and Public Financing

Reimbursement policies in Bulgaria and North Macedonia differ regarding- the structure of positive lists, patient co-payment rules, and pharmacy remuneration mechanisms (Table 3). Table 3 summarises reimbursement frameworks, including positive drug lists, reimbursement levels, patient co-payments, and contractual arrangements between pharmacies and health insurance funds.

In Bulgaria, reimbursed medicines are included in a Positive Drug List organised into multiple annexes, with varying levels of public funding [24,34]. Reimbursement decisions are closely linked to pricing regulation and, for selected products, health technology assessment. Pharmacies dispensing fully reimbursed medicines receive regulated remuneration subject to upper limits.

In North Macedonia, reimbursement is limited to medicines included in the national positive list and financed by the Health Insurance Fund. Patient co-payments are applied on a tiered basis and are capped both as a percentage of the reference price and in absolute monetary terms. Pharmacies receive fixed remuneration per dispensed prescription, reflecting a different approach to funding pharmaceutical services [35].

In addition to the Positive List, North Macedonia implements a mandatory list of Generics without Co-Payment (GLBD) [36]. This list includes reimbursed generic medicines for which patients do not pay additional fees, providing enhanced financial protection. Pharmacies are legally required to display the GLBD list in a visible place to ensure patient awareness and transparency. This mechanism is specific to North Macedonia and does not have a direct equivalent in Bulgaria.

### 3.4. Digitalisation of Prescribing and Dispensing Systems

Table 4 presents the level of digitalisation in prescribing, dispensing, and reimbursement processes.

The results indicate a higher degree of digital integration in Bulgaria, particularly with respect to electronic prescriptions for selected therapeutic categories and controlled substances. Digital registers and reporting systems support regulatory oversight and reimbursement processes.

In North Macedonia, digitalisation is progressing gradually through web-based prescribing and reporting systems linked to the Health Insurance Fund. However, printed prescriptions with manual authentication remain mandatory, indicating a hybrid system combining digital and paper-based elements. In North Macedonia, digitalisation is progressing gradually through electronic prescribing and reporting systems linked to the Health Insurance Fund. However, printed prescriptions with manual authentication remain in use for acute conditions, indicating a hybrid system combining digital and paper-based elements [23].

### 3.5. Financial Accessibility for Patients

Table 5 compares key factors influencing financial accessibility, including value-added tax (VAT) on medicines, patient co-payments, and affordability-related policy measures.

A notable difference between the two countries is the VAT rate applied to medicines, which is substantially lower in North Macedonia compared to Bulgaria. This contributes to lower final retail prices for patients, despite higher regulated pharmacy margins. In Bulgaria, the standard VAT rate applies to medicines, increasing out-of-pocket expenditure for patients, particularly for non-reimbursed products. Additional policy measures aimed at improving access include financial stimulations for pharmacies in underserved areas and the use of mobile pharmacies in North Macedonia, as well as targeted reimbursement mechanisms and expenditure control policies in Bulgaria.

## 4. Discussion

This comparative case study demonstrates that access to medicines in Bulgaria and North Macedonia is shaped by the combined effects of prescribing regulation, pricing mechanisms, reimbursement design, digitalisation, and fiscal policy, rather than by individual policy instruments alone. Similar findings have been reported in international analyses emphasising the systemic nature of pharmaceutical access policies [1,2]. Both countries operate under strict legislative control of prescribing and dispensing, consistent with international standards for medicine safety and rational use [3]. However, Bulgaria’s expanded use of mandatory electronic prescribing for selected therapeutic groups represents a move toward greater regulatory centralisation and monitoring, particularly for medicines with high public health or budgetary impact, such as antibiotics and antidiabetic agents [7]. North Macedonia’s model, combining paper prescriptions with digital reporting, provides operational flexibility but limits the full regulatory and analytical benefits of end-to-end electronic systems. Previous studies have shown that partial digitalisation may reduce efficiency gains and delay improvements in prescribing oversight [40]. These differences highlight electronic prescribing as both a technical and governance instrument.

Moreover, the use of external reference pricing in both countries aligns with WHO and EU recommendations for controlling medicine prices [4,5]. Nevertheless, differences in reference baskets, price calculation methods, and mark-up structures lead to divergent outcomes. Bulgaria’s manufacturer-price–based ceilings and capped margins reflect a cost-containment-oriented pricing model, consistent with policies in several EU Member States [9]. In contrast, North Macedonia’s higher percentage retail margins, particularly for low-priced medicines, prioritise pharmacy economic sustainability, a factor identified as critical for maintaining medicine availability in smaller markets [41]. These findings confirm that pricing regulation serves multiple policy objectives beyond affordability alone. Reimbursement policies in both countries are presented with positive lists and public health insurance financing, but differ substantially in structure. Bulgaria’s four-annex Positive Drug List and the integration of health technology assessment introduce additional mechanisms for expenditure control and prioritisation [8]. While effective for budget management, such complexity may increase administrative burden. North Macedonia applies a reimbursement framework, combining reference pricing with capped patient co-payments and fixed pharmacy remuneration per prescription. Similar models have been associated with improved predictability of patient payments and clearer separation between medicine prices and service remuneration [42].

Digitalisation plays an increasingly central role in pharmaceutical governance. Bulgaria’s broader implementation of electronic prescribing and digital registers enhances transparency, and reimbursement control, consistent with EU digital health priorities [43]. The COVID-19 pandemic further accelerated these reforms, reinforcing the role of digital tools in ensuring continuity of care [44]. North Macedonia’s gradual transition reflects institutional constraints common in health systems undergoing digital transformation [13]. Evidence suggests that incremental digitalisation can improve reporting and reimbursement processes, but full benefits are achieved only through system-wide integration [40].

Financial accessibility is strongly influenced by taxation and co-payment policies, in addition to price regulation. The lower value-added tax on medicines in North Macedonia contributes to reduced out-of-pocket expenditure, despite higher regulated retail margins. Similar associations between reduced VAT and improved affordability have been documented in other European contexts [45]. In Bulgaria, the application of the standard VAT rate increases the financial burden on patients, particularly for non-reimbursed medicines. Targeted measures, such as pharmacy incentives in underserved areas, partially mitigate access challenges but do not fully offset fiscal effects on affordability [46].

Although the present study primarily focuses on the comparative analysis of legislative and regulatory frameworks, the financial consequences of these policies are closely related to medicine price formation and pharmaceutical expenditure. The differences in price levels between Bulgaria and North Macedonia can be explained by differences in the principles of external reference pricing and the pricing base used in the two countries. Although both countries apply external reference pricing, they refer to different reference countries and use different methods for calculating the reference price. In Bulgaria, the price of medicinal products is determined based on the lowest manufacturer price among the reference countries. In contrast, in North Macedonia the reference price is calculated as the average of the two lowest prices, while the pricing base is the wholesale price.

Additionally, differences exist in terms of taxation. In Bulgaria, medicinal products are subject to the standard value-added tax (VAT) rate of 20%, whereas in North Macedonia a reduced VAT rate of 5% is applied to selected groups of medicines. This further contributes to lower final prices for some medicinal products in North Macedonia.

Future research incorporating quantitative comparisons of medicine prices, pharmaceutical expenditure, and patient cost-sharing would help further assess how these regulatory differences translate into measurable affordability outcomes. Future research could also examine differences in the composition of national positive drug lists and assess the real-world availability of medicines in community pharmacies, as these factors are important determinants of effective patient access to treatment.

The findings of this study contribute to the limited body of comparative research examining pharmaceutical policy frameworks in Southeast Europe, particularly between EU Member States and EU candidate countries. By systematically analysing legislative, pricing, reimbursement, and digitalisation mechanisms, the study provides evidence on how different policy instruments interact to shape financial access to medicines. These insights may support policymakers, health authorities, and regulatory institutions in identifying policy approaches that balance affordability, system sustainability, and equitable access to medicines. The comparative perspective may also inform future pharmaceutical policy reforms and contribute to ongoing discussions on regulatory harmonisation and health system strengthening in the region.

### Limitations and Policy Implications

The comparison between an EU Member State and an EU candidate country shows that regulatory alignment alone does not ensure similar access outcomes. Instead, national socioeconomic conditions and policy priorities shape the effectiveness of pharmaceutical regulation. Comparative evidence of this type can support policymakers in refining pricing, reimbursement, and digital health strategies to enhance equitable access while maintaining system sustainability [5,43].

This study has several limitations that should be considered when interpreting the findings. First, the analysis is based on documentary and legislative sources and does not include primary data collection, such as stakeholder interviews or patient-level quantitative data. As a result, the study focuses on policy design and regulatory frameworks rather than on measured health outcomes or patient experiences. Second, while the comparative case study approach allows for in-depth examination of national pharmaceutical systems, the findings are context-specific and may not be directly generalisable to other countries. However, the selected cases provide relevant insights for health systems with similar institutional characteristics or those undergoing regulatory alignment with European pharmaceutical policy frameworks. Additionally, the study relies on publicly available legal and policy documents, which may not fully capture differences in practical implementation, enforcement, or regional variation within each country. Differences in the operationalisation of prescribing rules, reimbursement criteria, and digital systems at the local level could not be systematically assessed. Finally, the analysis does not evaluate clinical effectiveness, patient adherence, or health outcomes, nor does it quantify the economic impact of pricing and reimbursement policies. Future research incorporating quantitative data and mixed-methods approaches could complement the present findings. Future research could complement the present policy analysis with quantitative comparisons of medicine prices, pharmaceutical expenditure, and patient out-of-pocket payments in order to further assess how regulatory differences translate into measurable affordability outcomes.

## 5. Conclusions

The study findings demonstrate that access to medicines is determined by the interaction of multiple regulatory and financial instruments, rather than by isolated policy measures. Despite the application of similar core principles, such as external reference pricing and positive reimbursement lists, the two countries exhibit distinct access outcomes. Bulgaria’s more extensive digitalisation of prescribing and reimbursement enhances regulatory oversight and system control but is accompanied by higher patient out-of-pocket expenditure, partly due to the application of the standard value-added tax on medicines. In contrast, North Macedonia combines lower taxation with capped co-payments and higher regulated pharmacy margins, contributing to improved financial affordability for patients while supporting pharmacy sustainability. The analysis also highlights the growing importance of digital health systems. The study contributes comparative evidence relevant to ongoing pharmaceutical policy reforms and supports cross-country learning aimed at improving equitable access to medicines while ensuring the sustainability of pharmaceutical systems.

## Figures and Tables

**Table 1:** Comparison of Prescribing and Dispensing Rules in Bulgaria and North Macedonia.

Aspect	Bulgaria	North Macedonia
Prescribing authority	Physicians, dentists, veterinarians; limited prescribing by physician assistants and feldshers under regulation [20]	Licensed physicians with a contract with the Health Insurance Fund for primary health care; prescriptions limited to their area of specialization Dentists—allowed to prescribe medications relevant to dental care [21]
Prescription format	Electronic prescriptions mandatory for selected therapeutic groups medicines (antidiabetic medicines and systemic antibiotics); paper prescriptions still permitted for others [20]	Prescriptions are mainly issued through web-based systems, while paper prescriptions are still required for acute treatment [22]
Prescription validity—acute treatment	Up to 30 days, depending on prescription type [20]	3 days from date of issue; treatment up to 8 days [22]
Prescription validity—chronic treatment	Repeat prescriptions valid up to 6 months [20]	Prescriptions may cover therapy up to 180 days with monthly dispensing [22]
Controlled substances	Electronic prescriptions with enhanced control and reporting [20]	Require both electronic and paper prescriptions for reimbursement; private dispensing is paper-based only (duplicate with enhanced control) [22]
Dispensing rules	Dispensing only by licensed pharmacists in community pharmacies [23]	Dispensing by pharmacists; generic substitution allowed with patient consent [22]

**Table 2:** Comparison of Pricing Mechanisms for Medicinal Products in Bulgaria and North Macedonia.

Pricing Process	Bulgaria	North Macedonia
Legal basis	The Medicinal Products in Human Medicine Act [24]; The Ordinance on the Terms, Rules and Procedure for Regulation and Registration of Prices for Medicinal products [24]	Law on Medicinal Products and Medical Devices [25]; Methodology for the Formation of Medicine Prices [26]
Pricing authority	National Council on Prices and Reimbursement of Medicinal Products (NCPRMP)	Ministry of Health (policy oversight) and Health Insurance Fund (determination of maximum reimbursed medicine prices), based on the Law on Health Insurance and the Law on Medicines and Medical Devices
Price regulation scope	Medicinal products reimbursed with public funds and included in the Positive Drug List (PDL) [24,26]	Medicinal products reimbursed by the Health Insurance Fund and included in the Positive List [27]
External reference pricing	Maximum manufacturer price set at the lowest reference prices in the 10 EU Member States [24]	Maximum price based on the average of the two lowest prices among reference countries [28]
Reference countries	Belgium, Greece, Spain, Italy, Latvia, Lithuania, Romania, Slovakia, Slovenia, France [24]	Slovenia, Croatia, Bulgaria, Serbia, Greece
Wholesale mark-up	Regulated percentage mark-up based on manufacturer’s price with upper monetary limits	Regulated percentage mark-up based on wholesale price
Retail mark-up	Regulated percentage mark-up based on manufacturer price with capped maximum values (ceiling prices of medicinal products)	Regulated percentage mark-up based on wholesale price, higher margins for low-priced medicines
VAT on medicines	Standard VAT rate of 20% applied to medicinal products [29]	Reduced VAT rate of 5% applied to medicinal products [30]
Prices of non-reimbursed medicines	Registered maximum retail prices without fixed wholesale or retail mark-ups [24]	Freely formed prices outside the Positive List

**Table 3:** Comparison of Reimbursement Policies in Bulgaria and North Macedonia.

Reimbursement Element	Bulgaria	North Macedonia
Legal basis	Health Insurance Act [31]; The Medicinal Products in Human Medicine Act [25]; Ordinance on the terms, rules and procedure for regulation and registration of prices for medicinal products [25]	Law on Health Insurance [32]; Law on Medicinal Products and Medical Devices [26]; Rulebook on Reference Prices [33]
Competent authority	National Health Insurance Fund (NHIF); National Council on Prices and Reimbursement of Medicinal Products	Reimbursement decisions are governed by the Ministry of Health, while the Health Insurance Fund is responsible for financing and implementation
Positive drug list	Positive Drug List (PDL) structured in four annexes according to funding source and reimbursement level	Positive List of Medicines reimbursed by the Health Insurance Fund [28]
Eligibility for reimbursement	Medicines included in the PDL and prescribed for indications covered by NHIF criteria [34]	Medicines included in the Positive List and prescribed to insured persons
Reimbursement level	Partial or full reimbursement depending on annex and therapeutic indication	Reimbursement based on reference price defined by the Health Insurance Fund
Patient co-payments	Variable co-payment depending on reimbursement level; no co-payment for selected fully reimbursed medicines	Tiered co-payment system capped at a maximum of 20% of the reference price and an absolute monetary ceiling [27]
Pharmacy remuneration	Regulated remuneration based on percentage of reimbursed medicine price with upper monetary limits	Product-based remuneration through regulated retail margins; dispensing remuneration embedded in reimbursement paid by the Health Insurance Fund; no fee-for-service for professional services
Settlement and payment	Pharmacies reimbursed by NHIF based on submitted prescriptions and contractual arrangements [34]	Pharmacies reimbursed by the Health Insurance Fund based on invoicing and prescription reporting [35]

**Table 4:** Digitalisation of Prescribing, Dispensing, and Reimbursement Processes in Bulgaria and North Macedonia.

Digitalisation Element	Bulgaria	North Macedonia
Legal framework for digital health	Medicinal Products in Human Medicine Act; Health Act; Ordinance No. 4 of 4 March 2009 on the Conditions and Procedure for Prescribing and Dispensing Medicinal Products	Law on Health Care; Law on Medicinal Products and Medical Devices; secondary legislation on e-health systems
Electronic prescribing	Mandatory electronic prescriptions for selected therapeutic groups (e.g., antidiabetic medicines, systemic antibiotics) and controlled substances	Electronic prescribing implemented through the Health Insurance Fund system; printed prescriptions are primarily used for acute conditions [22]
Prescription authentication	Electronic identification and digital signatures within national e-health systems	Electronic prescription authentication within the Health Insurance Fund system; printed prescriptions for acute conditions require physician signature and stamp
Dispensing documentation	Electronic verification and reporting through national health information systems	Dispensing based on electronic prescriptions, with electronic reporting to the Health Insurance Fund; paper prescriptions used for acute conditions
Digital registers	Centralized National Health Information System (NHIS) [37] for prescriptions, reimbursed medicines, and controlled substances	Electronic databases maintained by the Health Insurance Fund and medicines agency
Reimbursement reporting	Mandatory electronic submission of dispensed prescriptions to the National Health Insurance Fund	Electronic invoicing and reporting of dispensed medicines to the Health Insurance Fund
Role of digitalisation during COVID-19	Accelerated implementation of electronic prescribing to ensure continuity of care for chronic patients	Expansion of digital reporting and prescribing support systems during the pandemic

**Table 5:** Financial accessibility indicators (VAT, co-payments, incentives, affordability measures).

Financial Accessibility Element	Bulgaria	North Macedonia
Value-added tax (VAT) on medicines	Standard VAT rate of 20% applied to medicinal products	Reduced VAT rate of 5% applied to selected medicinal products
Out-of-pocket patient payments	Co-payments depend on reimbursement level; full payment for non-reimbursed medicines	Tiered co-payment system capped at 20% of the reference price and a fixed maximum amount
Affordability of non-reimbursed medicines	Higher final retail prices due to standard VAT and regulated price ceilings	Lower final prices supported by reduced VAT despite higher retail margins
Pharmacy remuneration and incentives	Regulated remuneration with additional financial incentives for pharmacies in underserved areas	Higher regulated retail margins and reimbursement-based pharmacy remuneration
Geographical access measures	Financial stimulation for pharmacies in small or remote settlements and underserved areas [38]	Pharmaceutical stations for ≥3500 residents; mobile pharmaceutical stations planned for <1000 residents; owners of multiple pharmacies must open one in a rural area [39]
Cost-containment measures	Expenditure caps, health technology assessment, and risk-sharing agreements	Reference pricing and capped co-payments to limit patient financial burden

## Data Availability

No new data were created or analyzed in this study. Data sharing is not applicable to this article.

## Abbreviations

The following abbreviations are used in this manuscript:

EU	European Union
NHIS	National Health Information System
PDL	Positive Drug List
VAT	Value-added tax
WHO	World Health Organization
